# Lipid Quality Changes in French Fries, Chicken Croquettes, and Chicken Nuggets Fried with High-Linoleic and High-Oleic Sunflower Oils in Domestic Deep Fryers

**DOI:** 10.3390/foods13152419

**Published:** 2024-07-30

**Authors:** María-Victoria Ruiz-Méndez, Joaquín Velasco, Adriana Salud Lastrucci, Gloria Márquez-Ruiz

**Affiliations:** 1 Instituto de la Grasa (IG), Consejo Superior de Investigaciones Científicas (CSIC), Campus Bd 46, Ctra. de Utrera km 1, 41013 Sevilla, Spain; jvelasco@ig.csic.es (J.V.);; 2 Instituto de Ciencia y Tecnología de los Alimentos y Nutrición (ICTAN), Consejo Superior de Investigaciones Científicas (CSIC), José Antonio Novais, 10, 28040 Madrid, Spain; gmarquez@ictan.csic.es

**Keywords:** frying, high-linoleic sunflower oil, high-oleic sunflower oil, French fries, nuggets, croquettes, polar compounds, sterols, squalene, tocopherols

## Abstract

The quality of fried products greatly depends on the changes occurring during frying. The purpose of this work was to study the lipid quality changes taking place in selected frozen foods after domestic deep-frying. Conventional, high-linoleic sunflower oil (HLSO) and high-oleic sunflower oil (HOSO) were used, and the frozen foods selected were French fries, croquettes, and nuggets. The foods were fried in domestic fryers under discontinuous conditions. Analyses included fatty acid composition, sterols, tocopherols, squalene, and lipid alteration levels. In all fried foods, the content of lipids increased after frying, which is consistent with previous findings. However, the lipid exchange between the food and the oil greatly depended on the food characteristics. Specifically, the levels of frying oil in the food lipids were about 90, 40, and 58% for French fries, croquettes, and nuggets, respectively. The main results obtained showed that lipid alteration levels considerably decreased and amounts of sterols and tocopherols significantly increased in French fries’ lipids after frying. In both chicken products, croquettes and nuggets, the best quality improvement observed was a significant decrease in cholesterol in food lipids due to the lipid exchange. Overall, frying with HLSO and HOSO improved the quality and nutritional properties of all products tested.

## 1. Introduction

Lipid changes occurring during frying, which impact the quality of fried products and their nutritional properties, continue to be of significant interest to consumers and the food industry, as highlighted in recent reviews [1,2,3]. Some of the most relevant studied aspects about lipid changes in frying include limiting the formation of undesirable oxidation compounds, determining the mechanisms of fat uptake, and optimizing frying technologies to reduce oil content. To produce high-quality fried products, it is desirable that they exhibit minimal lipid oxidation and polymerization, a favorable ratio of monounsaturated-to-saturated fatty acids, and contain minor lipid compounds with potential health benefits, known as bioactive compounds.

The incorporation of bioactive compounds from frying oils into foods is currently an emerging topic in frying, albeit one that has been less studied thus far. Most research in this area has predominantly focused on oils extracted from olives [4,5,6,7]. These studies concluded that olive oils and olive-pomace oils enhanced the nutritional value of fried products by incorporating polyphenols, phytosterols, tocopherols, squalene, and triperpenic alcohols. 

In this study, we selected conventional high-linoleic sunflower oil (HLSO) and high-oleic sunflower oil (HOSO) to investigate the changes in lipid quality resulting from the domestic frying of three types of frozen foods. HLSO and HOSO are among the oils most widely used for frying; the latter is extracted from sunflower seeds obtained from mutagenesis and breeding strategies in order to increase oleic acid content [8]. The frying performance of HLSO compared to HOSO has been extensively studied regarding chemical changes and oil degradation [8,9,10,11]. It is widely accepted that HOSO is more resistant to thermoxidative alterations due to its higher content of monounsaturated fatty acids. 

Sunflower oils contain several groups of minor compounds of nutritional interest, namely tocopherols, sterols, and squalene. 

Tocopherols are the major natural antioxidants in sunflower oils and among them α-tocopherol, the main form of vitamin E, is the most abundant. Tocopherols offer potential health benefits such as anti-inflammatory, anti-angiogenic, antiproliferative, and pro-apoptotic effects. Additionally, tocopherols may positively impact human health by modulating signal transduction and gene expression related to inflammation and immune system disorders [12]. The potent antioxidant action of tocopherols during frying has been well demonstrated and, although they are partly lost at frying conditions, the remaining amounts in the fried foods continue to protect them from oxidation during commercialization and storage [13]. 

Among plant sterols in general, and in sunflower oils in particular, β-sitosterol is the most abundant. Some clinical and preclinical studies indicate that β-sitosterol offers substantial health benefits by lowering LDL-cholesterol and preventing certain types of cancer [14]. β-sitosterol has also been reported to improve the frying stability of oils, although their antioxidant mechanism of action is not clear [15,16]. It has been proposed that conjugation of β-sitosterol into steradienes may be responsible for the antipolymerization effect observed [16].

Squalene is a bioactive compound that has shown positive effects; it is regarded as an anticarcinogenic, hypocholesterolemic, and detoxifying agent [17]. Under frying conditions, squalene has shown a moderate protective effect [18]. However, even though it has been suggested that squalene may regenerate α-tocopherol from the tocopheroxyl radical at low temperature [19], the mechanism of action at frying conditions remains unknown.

The frozen food products chosen for this study were among the most widely consumed and comprised both plant-based and animal-derived items. This selection was also based on their varying characteristics in terms of coating and/or pre-fried treatments. The products tested included pre-fried stick potatoes (commonly referred to as French fries), pre-fried battered chicken nuggets, and breaded chicken croquettes. These foods were fried in domestic fryers under discontinuous conditions, simulating typical frying processes used at home, as well as in restaurants and fast-food outlets. 

The oils and lipids extracted from the food products, both before and after frying, were analyzed for characterization and quality. Additionally, the levels of sterols, tocopherols, and squalene were quantified.

## 2. Materials and Methods

### 2.1. Chemicals

Standards of 5-α-Cholestan-3-ol, squalane, and tocopherols (α, β, γ, δ) with a purity of 99% were provided by Sigma-Aldrich SA (St. Louis, MO, USA). Chemicals and reagents (analytical grade) were purchased from local suppliers. 

### 2.2. Samples

HLSO (Muchel, Aceites Urzante, Tudela, Spain) and HOSO (Muchel, Aceites Urzante, Tudela, Spain) were obtained from a local market. Frozen French fries (Crinkle Fries McCain), frozen chicken croquettes (Makrochef), and frozen chicken nuggets (Makrochef) were purchased in Makro, Spain.

### 2.3. Frying Experiments

Three domestic deep fryers (Moulinex Easy Pro, Model F59-M, Groupe SEB, Lyon, France), each with a 3 L oil capacity, were utilized. Each fryer was used to fry four batches of 100 g frozen foods. The oil-specific surface in the fryers was 0.13 cm^−1^. Temperature control was managed with a K-type thermocouple connected to a recorder, ensuring that frying began at 175 ± 3 °C. The oil was initially heated for 5 min before frying commenced. After each frying session, the basket was shaken and allowed to drain for 1 min to remove excess oil.

Foods were fried during 10 min with 2-h intervals between frying sessions to replicate discontinuous frying conditions, following an established protocol [7]. The experiments were conducted in triplicate without adding fresh oil throughout the process. The total duration of the heating period for the oils was 6 h and 45 min. Samples of frying oil and fried foods were stored in a nitrogen atmosphere at −18 °C until they were analyzed. 

### 2.4. Analytical Procedures

#### 2.4.1. Oil Analyses

The acidity was determined using titration according to ISO 660:2020 method [20]. The peroxide value was determined using the iodometric assay following ISO 3960:2017 method [20]. The oil stability index was determined using a Rancimat device (Rancimat 743 equipment, Metrohm, Switzerland) at 110 °C following AOCS Official Method Cd-12b-92 [21]. The smoke point was measured according to AOCS Official Method Cc-9a-48 [21]. 

The fatty acid composition was analyzed using gas chromatography after derivatization to fatty acid methyl esters using 2M KOH in methanol, following IUPAC Standard Methods 2.301 and 2.302 [22]. 

Squalene was analyzed using gas chromatography according to AOCS Official Method Ch-9-02 [21], using squalane as an internal standard. 

Individual and total sterol contents were determined using gas chromatography following ISO 12228-1:2014 method [20] and 5-α-cholestan-3-ol was used as internal standard. 

Tocopherols were analyzed using high-performance liquid chromatography with fluorescence detection according to IUPAC Standard Method 2.432 [22]. Tocopherol standards were used for external calibration.

Total polar compounds were separated and quantified using silica column chromatography following IUPAC method 2.507 [22]. The fractions of polar compounds were then analyzed using HPSEC-RID, as reported elsewhere [23], to quantitate oxidized triacylglycerols, polymers (dimers and higher oligomers), mono- and di-acylglycerols, and free fatty acids.

#### 2.4.2. Food Analyses

(a) Lipid content

One hundred grams of initial and fried foods were frozen at −40 °C for 24 h, then freeze-dried in a Heto FD3 freeze-dryer (Heto-Holten A/S, Allerod, Denmark) at −50 °C and 10^−2^ mmHg for about 48 h, until constant weight. Samples were then ground, and the total lipids were extracted with hexane in a Söxhlet extractor for 6 h, following UNE 55-062-80 method [24].

(b) Food lipid analyses

The lipids extracted from the foods were also analyzed according to Section 2.4.1.

### 2.5. Statistical Analyses

The frying tests were conducted in triplicate. The results are presented as mean values with standard deviations of three analyses in independent samples. The student’s *t*-test was used to compare two sets of data, while ANOVA followed by Tukey’s test was employed for comparing three or more sets of data. The statistical differences were considered significant at a level of *p* < 0.05. The SPSS 27 statistical program (IBM) was used for the analyses.

## 3. Results and Discussion

### 3.1. Characterization and Quality of the Oils

The fatty acid compositions of the oils used in this study were within the range usually found [8] and showed, consistent with previous findings, a high proportion of oleic acid in HOSO, whereas linoleic acid was the most abundant fatty acid in HLSO (Table 1).

Quality parameters, namely, acidity, peroxide value, and polar compounds, including the specific values for the oxidation and hydrolysis groups of compounds, were within the values characteristic of good quality refined oils (Table 1). HLSO and HOSO greatly differed in oxidative stability index values, consistent with their distinct degree of unsaturation. The oxidative stability index allows for the ranking of oils according to their oxidative resistance at low and moderate temperatures, although it does not predict the oil stability during frying. As to smoke points, values for HLSO and HOSO were 55 °C and 56 °C, respectively, above the temperature used for frying in this study (175 ± 3 °C).

Regarding minor bioactive components, both HLSO and HOSO presented high contents of tocopherols, with α-tocopherol being predominant, accounting for 89.7% and 91.1%, respectively (Table 1). These values are consistent with published data, as well as the squalene and sterols levels found in HLSO and HOSO [8].

### 3.2. Changes in Food Lipid Contents during Frying

In line with previous findings, the foods underwent an increase in the lipid content during frying, but the extent of this increase varied significantly among them (Table 2). French fries and croquettes showed the highest increment, nearly doubling their lipid content. However, the lipid increase in nuggets was below 20%. It is widely recognized that variables such as product shape, porosity, food surface structure and composition, moisture and lipid contents, and pre-frying treatments play crucial roles in determining the composition of the finished fried product [25]. The results showed that there were no significant differences in the lipid content between products fried with different oils. In this regard, a debated question remains whether the degree of oil unsaturation influences oil uptake, with contradictory results obtained so far [1,26,27].

### 3.3. Changes in the Fatty Acid Composition of the Food Lipids

The lipid extract of initial French fries contained similar levels of oleic and linoleic acids and a considerable proportion of stearic acid (Table 3). This fatty acid profile seemed to correspond to a mixture of vegetable oils. In fact, the product label stated that French fries were pre-fried with one or more of the following oils: canola, soybean, cottonseed, sunflower, and corn.

The fatty acid compositions of the French fries’ lipid extracts showed significant differences after frying. This was particularly evident in the great changes in stearic, linoleic, and oleic acids contents, attributed to the absorption of frying oil and lipid exchange (Table 3). When the fatty acid composition of the food lipids is substantially different from that of the oil, the extent of lipid exchange can be estimated by analyzing specific fatty acids, especially those whose differences exceed 30% [7]. Specifically, considering a particular fatty acid, the percentage of frying oil in fried food lipids was calculated using the following equation:

$$Frying\;oil\;\%\;in\;fried\;food\;lipids=\left(1-\frac{\%{FA}_{\mathrm{FF}}-\%{FA}_{\mathrm{FO}}}{\%{FA}_{\mathrm{IF}}-\%{FA}_{\mathrm{FO}}}\right)\;100$$

where %*FA_FF_*, %*FA_FO_*, and %*FA_IF_* represent the percentage of the fatty acid in the fried food lipids, frying oil, and initial food lipids, respectively. 

Based on changes in oleic acid percentages, the estimated proportions of frying oil in the fried French fries’ lipids were 90.3 and 92.3%, respectively. This highlights that the lipid quality and composition of fried French fries are primarily determined by those of the frying oil used, as already reported [25]. The extensive oil exchange observed in French fries can be attributed to the location of the oil absorbed during pre-frying, primarily on the surface layers of the food. Thus, food lipids can easily be released into the frying oil and exchange with it during the frying process.

The fatty acid composition of the lipid extracts from French fries was very similar to that of the oil used, HLSO or HOSO, albeit specific fatty acid values were generally found to be significantly different. 

The croquettes used in this study, as stated on the product label, were basically made of chicken mixed with wheat flour, milk, and broth, and coated with wheat breadcrumbs. The fatty acid composition of this product considerably changed after frying. Thus, great differences were found for virtually all fatty acids (Table 4). In addition, significant differences were also found between the fatty acid composition of the fried foods and those of the frying oils.

The proportion of frying oil in croquettes, estimated from changes in stearic acid percentages, indicated that 43.7 and 38.6% of the lipid content in croquettes fried with HLSO and HOSO, respectively, came from the frying oil. Nevertheless, variable results can be obtained in these products since oil uptake is greatly conditioned by the size, shape, and composition of croquettes, and even the particle size of the flour used can have a significant effect [28]. 

Frozen chicken nuggets were introduced by the fast-food industry and are considered one of the most successful products of the poultry industry [29]. Therefore, there is growing interest in various aspects of chicken nugget production, including the optimization of frying conditions to enhance their physicochemical and textural properties [30,31] and studying the kinetics of mass transfer during frying [32]. The products used in this study were pre-fried with unspecified vegetable oil and had a high lipid content (Table 2), as compared to data available on commercialized nuggets [29].

Like the croquettes, the fatty acid composition of the nuggets significantly changed after frying. Significant differences were observed in major fatty acids (Table 5). Also, significant differences were found between the fatty acid composition of the nuggets’ lipids and that of the frying oils at the end of the trial.

The proportion of frying oil in the fried nuggets, estimated from changes in oleic acid percentages, was 59.1% and 58.3% for HLSO and HOSO, respectively. Like French fries, chicken nuggets are pre-fried products, but they showed a much lower lipid exchange, likely due to their batter coating.

### 3.4. Changes in Polar Compounds

It is important to note that significantly higher amounts of polar compounds were formed in HLSO as compared to HOSO in all frying trials (Table 6), despite both oils initially showing similar, low levels (Table 1). These results agree with those normally obtained [8,9,10,11]. Nevertheless, the levels reached after four discontinuous frying operations, ranging from 15.0% to 16.9%, were far below the rejection limit for human consumption set by most countries that regulate frying fats and oils (25%) [33]. The increase in polar compounds primarily resulted from the formation of oxidized and polymeric compounds. 

Because of oil absorption and lipid exchange, all products fried with HLSO showed significantly higher levels of polar compounds than those fried with HOSO. In the case of French fries, initially containing levels of polar compounds surpassing the rejection limit, the quality greatly improved after frying, even with HLSO. The results also showed that, in the French fries’ trial, the total polar compounds and their composition did not significantly differ between the final product lipids and the final frying oils. However, this was not observed in croquettes and nuggets. This is another consequence of the notably higher lipid exchange that specifically occurred with French fries.

### 3.5. Changes in Sterols, Tocopherols and Squalene in the Food Lipids

A significant decrease in the total concentration of sterols was found in French fries’ lipids because of frying (Table 7). This phenomenon can be attributed to the significantly lower levels of sterols in both SO and HOSO (Table 1) and the high proportion of frying oils absorbed by the food (estimated to be over 90%). No significant differences were observed in the total sterols and their specific compounds between the final French fries’ lipids and the final frying oils. In both initial French fries’ lipids and initial oils, β-Sitosterol was the most abundant sterol, and this remained consistent in the final French fries’ lipids and final frying oils. 

Based on the data obtained, it was not possible to determine the extent of sterol degradation during frying, but significant losses were likely to have occurred [7,34,35].

Cholesterol from chicken fat was the major sterol in croquettes, and its proportion significantly decreased after frying with either HLSO or HOSO (Table 8). It was also observed that the total amount of sterols was lower in the fried croquettes’ lipids, although this was only significant when using HOSO. This indicates an improvement in the nutritional quality of the products, as reducing cholesterol in food lipids is highly recommended. 

A significant decrease in the proportion of cholesterol was also found in the nuggets’ lipids after frying, along with an increase in the proportion of β-sitosterol (Table 9). However, similar or even higher amounts of total sterols were found in the final lipid extracts compared to the initial food.

**Figure 1:**
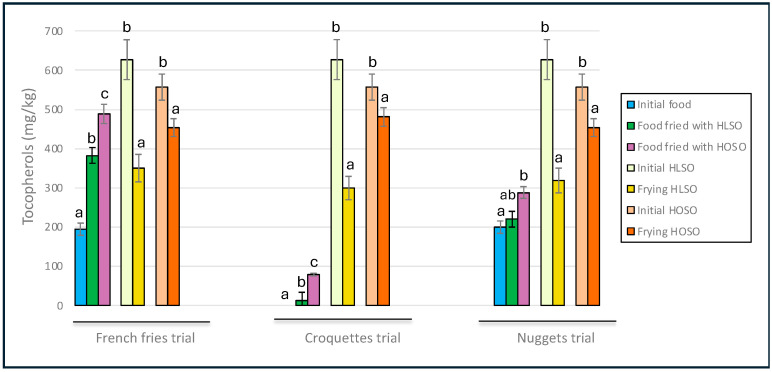
Tocopherols contents in initial and final food lipids and oils. Abbreviations: HLSO, high-linoleic sunflower oil; HOSO, high-oleic sunflower oil. Means ± SD (*n* = 3). Different letters in lipid foods and oils within each trial indicate significant differences (*p* < 0.05).

Tocopherol levels generally increased in all products because of frying, especially in French fries (Figure 1). Concurrently, great losses of tocopherols, because of their rapid degradation at frying temperatures and their antioxidant action [36], were found in both HLSO and HOSO.

The content of tocopherols is considered a relevant nutritional parameter in fried foods. Several studies have demonstrated a substantial enrichment of tocopherols in French fries during frying [4,7,25,37]. Evidently, this enrichment largely depends on the tocopherol content in the frying oil used and the degree of oil degradation during the process. Based on the data obtained in this study, it was calculated that just half a portion (100 g) of French fries would provide 8.5 or 11.2 mg tocopherols, covering up to 75% of the recommended daily intake of 15 mg vitamin E for adults [38]. In croquettes and nuggets, only frying with HOSO resulted in a low but significant enrichment of tocopherol. To the best of our knowledge, only two studies have reported on tocopherol changes in nuggets during frying [6,7]. Neither significant changes were found after four cycles of deep-frying with extra virgin olive, grapeseed, and canola oils [6], nor with olive-pomace oils [7].

**Figure 2:**
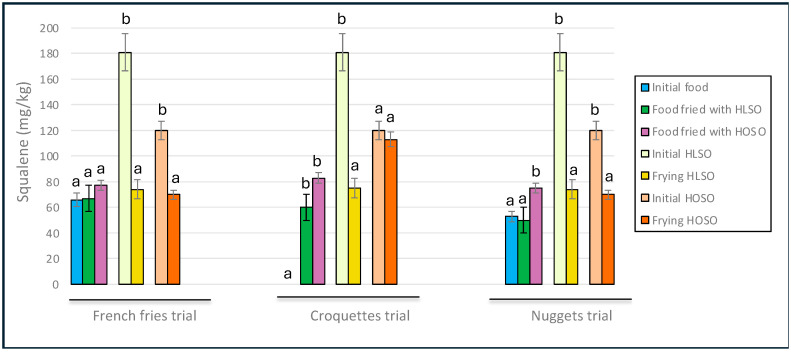
Squalene contents in initial and final food lipids and oils. Abbreviations: HLSO, high-linoleic sunflower oil; HOSO, high-oleic sunflower oil. Means ± SD (*n* = 3). Different letters in lipid foods and oils within each trial indicate significant differences (*p* < 0.05).

Significant losses of squalene in the frying oils were generally observed (Figure 2), which is consistent with previous findings from frying experiments using olive-pomace oils where similar levels of oil alteration were reached [7]. 

Those experiments indicated that losses of squalene between 20% and 40% can occur during the frying process [7]. In this context, Kalogeropoulos and Andrikopoulos reported that the retention of squalene in various vegetable oils used for frying in restaurants was over 50% and even higher values were observed during the domestic frying of potatoes in virgin olive oils [39].

In the present study, the oils used contained low levels of squalene, which is expected in vegetable oils other than olive-extracted oils. However, a significant enrichment in squalene was specifically observed in the croquettes, which had none before frying.

## 4. Conclusions

This study presents new findings on the lipid exchange occurring in frying between HLSO or HOSO and three widely consumed commercial frozen foods, demonstrating significant changes in food lipid quality. The most notable improvements in the quality of food lipids achieved through frying are summarized as follows. French fries gained more lipids but showed a very high lipid exchange (over 90%) with the frying oils, resulting in relevant quality improvements. Thus, the levels of lipid alteration decreased considerably when either HLSO or HOSO was used. When HOSO was employed, the fatty acid composition improved notably, showing an increase in the ratio of monounsaturated-to-polyunsaturated fatty acids, which is considered beneficial for health. Also, the amounts of sterols and tocopherols increased significantly using either HLSO or HOSO. In croquettes and nuggets, lipid exchange was lower, about 40 and 58%, respectively. The main results showed that in both croquettes and nuggets, the initial levels of cholesterol in the food lipids, coming from chicken fat, were very high and decreased significantly through frying. Croquettes were the only product that showed a significant increase in squalene content. Overall, it is concluded that using good quality sunflower oils for frying frozen products can significantly enhance their quality and increase the content of bioactive compounds.

## Figures and Tables

**Table 1:** Characterization and quality parameters of the oils.

	HLSO	HOSO
Fatty acids (wt %)		
C14:0	0.08 ± 0.00	0.05 ± 0.00
C16:0	6.02 ± 0.00	3.73 ± 0.01
C16:1	0.09 ± 0.00	0.10 ± 0.00
C17:0	0.03 ± 0.00	0.01 ± 0.00
C17:1	nd	0.02 ± 0.00
C18:0	3.73 ± 0.01	3.09 ± 0.01
C18:1	29.31 ± 0.01	83.55 ± 0.02
C18:2	58.83 ± 0.01	7.58 ± 0.01
*Trans* C18:2	0.10 ± 0.00	0.02 ± 0.00
C18:3	0.06 ± 0.00	0.05 ± 0.00
C20:0	0.22 ± 0.01	0.24 ± 0.00
C20:1	0.14 ± 0.01	0.23 ± 0.01
C20:4	0.01 ± 0.00	0.01 ± 0.00
C22:0	0.57 ± 0.01	0.77 ± 0.01
C24:0	0.11 ± 0.01	0.25 ± 0.00
Others	0.70 ± 0.01	0.34 ± 0.01
Acidity (% oleic acid)	0.04 ± 0.01	0.07 ± 0.01
Peroxide value (meq O_2_/kg oil)	5.7 ± 1.0	3.2 ± 0.7
Oxidative stability index (h)	5.3 ± 0.3	22.1 ± 1.1
Smoke point (°C)	230 ± 2	234 ± 3
Polar compounds (wt %)		
Total	2.6 ± 0.1	2.3 ± 0.1
Triacylglycerol dimers	0.4 ± 0.0	0.1 ± 0.0
Oxidized triacylglycerols	1.1 ± 0.1	0.6 ± 0.1
Diacylglycerols	0.8 ± 0.1	1.3 ± 0.1
Monoacylglycerols	nd	nd
Free fatty acids *	0.3 ± 0.0	0.4 ± 0.1
Bioactive compounds (mg/kg)		
Tocopherols	627 ± 14	557 ± 8
Squalene	181 ± 8	120 ± 9
Sterols	3219 ± 25	3198 ± 18

Abbreviations: HLSO, high-linoleic sunflower oil; HOSO, high-oleic sunflower oil. Means ± SD (*n* = 3). * It also includes other polar minor oil components, nd: not detected.

**Table 2:** Lipid content in initial foods and foods fried in the fourth discontinuous frying operation.

Lipid Content (wt % on Food)	Initial	Fried with HLSO	Fried with HOSO
French fries	12.8 ± 0.6 a	22.3 ± 2.1 b	22.8 ± 1.1 b
Croquettes	21.4 ± 2.2 a	38.3 ± 2.6 b	39.1 ± 1.2 b
Nuggets	32.9 ± 1.1 a	38.5 ± 2.1 b	36.3 ± 3.4 b

Abbreviations: HLSO, high-linoleic sunflower oil; HOSO, high-oleic sunflower oil. Means ± SD (*n* = 3). Different letters in a row indicate significant differences (*p* < 0.05).

**Table 3:** Fatty acid compositions of the lipids extracted from French fries and those of frying oils after the fourth discontinuous frying operation.

Fatty Acids (wt % on Oil)	Initial Lipid Extract	HLSO		HOSO	
		Final Oil	Final Lipid Extract	Final Oil	Final Lipid Extract
C16:0	11.55 ± 0.03	6.32 ± 0.03 a	6.50 ± 0.06 b *	3.84 ± 0.00 a	4.28 ± 0.01 b *
C16:1	0.13 ± 0.01	0.09 ± 0.00 a	0.09 ± 0.00 a *	0.10 ± 0.00 a	0.10 ± 0.00 a *
C18:0	3.43 ± 0.01	3.87 ± 0.02 a	3.83 ± 0.02 a *	3.12 ± 0.00 a	3.12 ± 0.00 a *
C18:1	42.52 ± 0.06	30.22 ± 0.08 a	30.59 ± 0.13 b *	83.26 ± 0.03 b	80.4 ± 0.09 a *
C18:2	40.62 ± 0.09	57.47 ± 0.14 b	57.04 ± 0.22 a *	7.66 ± 0.01 a	10.00 ± 0.08 b *
*Trans* C18:2	0.14 ± 0.01	0.13 ± 0.01 a	0.12 ± 0.01 a	0.08 ± 0.10 a	0.02 ± 0.01 a *
C18:3	0.24 ± 0.02	0.06 ± 0.00 a	0.12 ± 0.01 b *	0.05 ± 0.00 a	0.13 ± 0.01 b *
C20:0	0.24 ± 0.01	0.25 ± 0.01 a	0.25 ± 0.01 a	0.26 ± 0.01 a	0.25 ± 0.01 a
C20:1	0.17 ± 0.01	0.10 ± 0.01 a	0.11 ± 0.01 a *	0.25 ± 0.00 a	0.25 ± 0.00 a *
C22:0	0.54 ± 0.03	0.71 ± 0.02 a	0.69 ± 0.02 a *	0.91 ± 0.00 b	0.88 ± 0.01 a *
C24:0	0.19 ± 0.01	0.22 ± 0.01 a	0.22 ± 0.01 a *	0.29 ± 0.00 a	0.28 ± 0.00 a *
Others	0.23 ± 0.01	0.56 ± 0.30 a	0.34 ± 0.13 a	0.05 ± 0.12 a	0.24 ± 0.01 b

Abbreviations: HLSO, high-linoleic sunflower oil; HOSO, high-oleic sunflower oil. Means ± SD (*n* = 3). Different letters in the same row indicate significant differences between the final lipid extract and the final frying oil for a given oil. Asterisks indicate significant differences between the initial and final lipid extracts for a given frying oil (*p* < 0.05).

**Table 4:** Fatty acid compositions of the lipids extracted from croquettes and those of frying oils after the fourth discontinuous frying operation.

Fatty Acids (wt % on Oil)	Initial Lipid Extract	HLSO		HOSO	
		Final Oil	Final Lipid Extract	Final Oil	Final Lipid Extract
C16:0	12.77 ± 0.12	6.33 ± 0.01 a	9.82 ± 0.62 b *	3.79 ± 0.00 a	9.28 ± 0.14 b *
C16:1	1.08 ± 0.01	0.09 ± 0.00 a	0.64 ± 0.05 b *	0.10 ± 0.00 a	0.86 ± 0.02 b *
C18:0	4.52 ± 0.08	3.90 ± 0.00 a	5.00 ± 0.32 b	3.15 ± 0.01 a	4.79 ± 0.06 b *
C18:1	54.00 ± 0.15	30.26 ± 0.05 a	34.50 ± 0.57 b *	83.66 ± 0.07 b	61.27 ± 0.58 a *
C18:2	24.29 ± 0.12	57.43 ± 0.02 b	47.64 ± 1.02 a *	7.40 ± 0.03 a	20.68 ± 0.29 b *
*trans* C18:1	0.16 ± 0.06	nd	nd	nd	0.14 ± 0.00
*trans* C18:2	0.07 ± 0.01	0.12 ± 0.01 a	0.12 ± 0.00 a *	nd	0.05 ± 0.00
C18:3	0.59 ± 0.01	0.06 ± 0.00 a	0.23 ± 0.00 b *	0.05 ± 0.00 a	0.26 ± 0.02 b *
C20:0	0.37 ± 0.00	0.25 ± 0.00 a	0.24 ± 0.01 a *	0.26 ± 0.00 a	0.25 ± 0.00 a *
C20:1	0.39 ± 0.01	0.17 ± 0.00 a	0.29 ± 0.01 b *	0.25 ± 0.01 a	0.38 ± 0.00 b
C22:0	0.02 ± 0.01	0.71 ± 0.00 b	0.25 ± 0.01 a *	0.89 ± 0.02 b	0.63 ± 0.01 a *
C24:0	0.11 ± 0.00	0.23 ± 0.01 b	0.20 ± 0.01 a *	0.28 ± 0.0 b1	0.20 ± 0.00 a *
Others	1.37 ± 0.23	0.45 ± 0.12 a	1.07 ± 0.08 b	0.01 ± 0.01 a	1.02 ± 0.05 b

Abbreviations: HLSO, high-linoleic sunflower oil; HOSO, high-oleic sunflower oil. nd: not detected. Means ± SD (*n* = 3). Different letters in the same row indicate significant differences between the final lipid extract and the final frying oil for a given oil. Asterisks indicate significant differences between the initial and final lipid extracts for a given frying oil (*p* < 0.05).

**Table 5:** Fatty acid compositions of the lipids extracted from nuggets and those of frying oils after the fourth discontinuous frying operation.

Fatty Acids (wt % on Oil)	Initial Lipid Extract	HLSO		HOSO	
		Final Oil	Final Lipid Extract	Final Oil	Final Lipid Extract
C16:0	14.25 ± 0.12	6.31 ± 0.02 a	13.06 ± 0.23 b *	12.21 ± 0.09 a	17.54 ± 0.10 b *
C16:1	2.75 ± 0.01	0.10 ± 0.01 a	2.30 ± 0.09 b *	0.90 ± 0.02 a	2.16 ± 1.85 a
C18:0	4.95 ± 0.08	3.90 ± 0.01 a	4.68 ± 0.02 b *	3.02 ± 0.09 a	4.23 ± 0.08 b *
C18:1	40.04 ± 0.15	30.38 ± 0.07 a	34.33 ± 0.11 b *	69.91 ± 0.29 b	57.44 ± 0.11 a *
C18:2	34.26 ± 0.12	57.41 ± 0.14 b	41.94 ± 0.99 a *	11.12 ± 0.30 a	13.81 ± 0.27 b *
*Trans* C18:1	0.16 ± 0.06	nd	nd	nd	nd
*Trans* C18:2	0.07 ± 0.01	0.12 ± 0.00 b	0.09 ± 0.01 a	0.04 ± 0.00 a	0.05 ± 0.01 b
C18:3	0.95 ± 0.01	0.06 ± 0.01 a	0.81 ± 0.01 b *	0.60 ± 0.02 a	0.76 ± 0.01 b *
C20:0	0.27 ± 0.01	0.22 ± 0.04 a	0.27 ± 0.01 a	0.50 ± 0.00 b	0.37 ± 0.00 a *
C20:1	0.04 ± 0.01	0.19 ± 0.05 a	0.26 ± 0.01 b *	0.37 ± 0.00 a	0.39 ± 0.01 b *
C22:0	0.04 ± 0.01	0.68 ± 0.01 b	0.03 ± 0.01 a	0.20 ± 0.00 b	0.03 ± 0.00 a
C24:0	0.12 ± 0.01	0.22 ± 0.01 b	0.15 ± 0.01 a *	0.08 ± 0.00 b	0.06 ± 0.00 a *
Others	2.10 ± 0.73	0.41 ± 0.10 a	2.08 ± 0.73 b	0.57 ± 0.06 a	2.66 ± 1.95 a

Abbreviations: HLSO, high-linoleic sunflower oil; HOSO, high-oleic sunflower oil. nd: not detected. Means ± SD (*n* = 3). Different letters in the same row indicate significant differences between the final lipid extract and the final frying oil for a given oil. Asterisks indicate significant differences between the initial and final lipid extracts for a given frying oil (*p* < 0.05).

**Table 6:** Polar compounds content and distribution of initial and fried food lipids and oils.

Polar Compounds (wt %)	Total	Oligomers	Dimers	Oxidized Triacylglycerols	Diacyl Glycerols	Monoacyl Glycerols	Free Fatty Acids
**French fries’ trial**							
Initial French fries extract	28.3 ± 0.2	6.9 ± 0.1	9.3 ± 0.1	8.6 ± 0.1	2.7 ± 0.1	nd	0.8 ± 0.0
French fries extract (fried with HLSO)	14.8 ± 1.3 a *	1.1 ± 0.3 a *	5.9 ± 0.9 a *	5.9 ± 0.4 a *	1.5 ± 0.1 b *	nd	0.5 ± 0.0 a *
Final frying HLSO	15.0 ± 1.6 a	1.2 ± 0.3 a	6.6 ± 1.0 a	5.6 ± 0.4 a	1.2 ± 0.0 a	nd	0.4 ± 0.0 a
French fries extract (fried with HOSO)	3.3 ± 0.8 a *	0.1 ± 0.0 a *	0.7 ± 0.3 a *	0.9 ± 0.2 a *	1.3 ± 0.3 a *	nd	0.3 ± 0.1 a *
Final frying HOSO	3.4 ± 0.8 a	0.1 ± 0.0 a	0.8 ± 0.2 a	1.0 ± 0.3 a	1.2 ± 0.3 a	nd	0.3 ± 0.1 a
**Croquettes trial**							
Initial croquettes extract	7.5 ± 0.4	0.2 ± 0.0	0.8 ± 0.1	4.6 ± 0.2	1.2 ± 0.0	nd	0.8 ± 0.1
Croquettes extract (fried with HLSO)	15.1 ± 0.9 a *	0.8 ± 0.1 a *	6.8 ± 0.3 a *	5.6 ± 0.4 a *	1.2 ± 0.8 a	nd	0.3 ± 0.1 a *
Final frying HLSO	16.9 ± 0.4 b	1.4 ± 0.0 b	7.2 ± 0.1 a	5.5 ± 0.2 a	2.5 ± 0.0 b	nd	0.3 ± 0.1 a
Croquettes extract (fried with HOSO)	5.4 ± 0.8 a *	0.3 ± 0.1 a	1.7 ± 0.2 b *	1.8 ± 0.1 a *	1.0 ± 0.1 a *	nd	0.6 ± 0.0 a *
Final frying HOSO	7.1 ± 0.4 b	0.4 ± 0.1 a	1.2 ± 0.1 a	2.4 ± 0.4 b	2.4 ± 0.3 b	nd	0.6 ± 0.0 a
**Nuggets trial**							
Initial nuggets extract	2.3 ± 0.1	nd	0.1 ± 0.0	0.6 ± 0.1	1.2 ± 0.2	nd	0.3 ± 0.1
Nuggets extract (fried with HLSO)	10.9 ± 1.4 a *	0.8 ± 0.1 a	4.3 ± 0.2 a *	4.0 ± 0.4 a *	1.1 ± 0.3 a	nd	0.8 ± 0.2 b *
Final frying HLSO	16.4 ± 0.9 b	1.4 ± 0.1 b	7.2 ± 0.3 b	4.9 ± 0.7 a	2.5 ± 0.4 b	nd	0.3 ± 0.1 a
Nuggets extract (fried with HOSO)	4.6 ± 0.2 a *	0.1 ± 0.0 a	0.7 ± 0.1 a *	1.2 ± 0.2 a *	1.7 ± 0.3 a	0.9 ± 0.0 a	0.1 ± 0.0 a *
Final frying HOSO	8.6 ± 0.3 b	0.2 ± 0.0 b	1.5 ± 0.1 b	2.4 ± 0.2 b	3.5 ± 0.3 b	0.9 ± 0.1 a	0.1 ± 0.0 a

Abbreviations: HLSO, high-linoleic sunflower oil; HOSO, high-oleic sunflower oil. nd: not detected. Means ± SD (*n* = 3). Different letters in the same column for a given frying oil indicate significant differences between the final food lipid extract and the final frying oil. Asterisks indicate significant differences between the initial lipid extracts and the final lipid extracts for a given frying oil (*p* < 0.05).

**Table 7:** Sterols composition and total content in lipids extracted from French fries and frying oils after the fourth discontinuous frying operation.

Sterols (wt % on Total)	Initial Lipid Extract	HLSO		HOSO	
		Final Oil	Final Lipid Extract	Final Oil	Final Lipid Extract
Cholesterol	0.59 ± 0.08	0.16 ± 0.05 a	0.31 ± 0.02 b *	0.25 ± 0.06 a	0.30 ± 0.08 a *
Brassicasterol	0.19 ± 0.08	0.03 ± 0.05 a	0.01 ± 0.02 a *	0.02 ± 0.04 a	0.01 ± 0.02 a *
Campesterol	7.47 ± 0.07	9.07 ± 0.03 b	8.95 ± 0.06 a *	9.05 ± 0.13 a	9.08 ± 0.35 a *
Campestanol	0.94 ± 0.10	0.15 ± 0.01 a	0.09 ± 0.08 a *	0.08 ± 0.09 a	0.07 ± 0.07 a *
Stigmasterol	7.48 ± 0.21	8.90 ± 0.00 b	8.45 ± 0.09 a *	8.70 ± 0.18 a	8.74 ± 0.31 a *
Δ7-Campesterol	2.08 ± 0.11	2.34 ± 0.08 b	2.02 ± 0.05 a	2.16 ± 0.13 a	2.40 ± 0.33 a
Δ5.23-Stigmastadienol	1.28 ± 0.23	0.86 ± 0.10 b	0.67 ± 0.04 a *	0.99 ± 0.12 a	0.94 ± 0.06 a
Clerosterol	2.83 ± 1.33	0.29 ± 0.33 a	0.34 ± 0.39 a *	1.00 ± 0.44 a	0.79 ± 1.05 a
β-Sitosterol	49.88 ± 1.90	58.16 ± 0.57 a	58.61 ± 0.67 a *	58.95 ± 1.14 a	58.48 ± 1.43 a *
Sitostanol	2.39 ± 0.24	1.17 ± 0.14 a	1.14 ± 0.06 a *	1.25 ± 0.05 a	1.20 ± 0.04 a *
Δ5-Avenasterol	4.18 ± 0.19	1.72 ± 0.02 a	2.57 ± 0.09 b *	1.12 ± 0.07 a	2.18 ± 0.06 b *
Δ5.24-Stigmastadienol	2.05 ± 0.61	2.46 ± 0.10 b	2.26 ± 0.07 a	3.07 ± 0.10 a	2.93 ± 0.13 a
Δ7-Estigmastenol	15.19 ± 0.72	10.94 ± 0.10 a	10.91 ± 0.28 a *	10.86 ± 0.62 a	10.32 ± 1.05 a *
Δ7-Avenasterol	3.09 ± 0.31	3.57 ± 0.08 a	3.44 ± 0.15 a	2.33 ± 0.16 a	2.42 ± 0.12 a *
Total sterols (mg/kg lipids)	3963 ± 100	3509 ± 78 a	3555 ± 70 a *	3393 ± 80 a	3570 ± 111 a *

Abbreviations: HLSO, high-linoleic sunflower oil; HOSO, high-oleic sunflower oil. Means ± SD (*n* = 3). Different letters in the same row indicate significant differences between the final lipid extract and the final frying oil for a given oil. Asterisks indicate significant differences between the initial and final lipid extracts for a given frying oil (*p* < 0.05).

**Table 8:** Sterols composition and total content of lipids extracted from croquettes and frying oils after the fourth discontinuous frying operation.

Sterols (wt % on Oil)	Initial Lipid Extract	HLSO		HOSO	
		Final Oil	Final Lipid Extract	Final Oil	Final Lipid Extract
Cholesterol	36.95 ± 0.47	1.57 ± 1.67 a	20.39 ± 1.32 b *	0.58 ± 0.11 a	23.13 ± 0.66 b *
Campesterol	6.85 ± 0.01	8.38 ± 0.08 b	7.75 ± 0.21 a *	8.87 ± 0.08 b	7.43 ± 0.22 a *
Stigmasterol	4.24 ± 0.07	6.97 ± 0.10 b	5.53 ± 0.09 a *	7.49 ± 0.16 b	5.48 ± 0.05 a *
Δ7-Campesterol	0.97 ± 0.01	2.09 ± 0.42 a	1.55 ± 0.26 a *	2.78 ± 0.19 b	1.62 ± 0.16 a *
Δ5.23-Stigmastadienol	0.37 ± 0.03	0.72 ± 0.13 a	0.61 ± 0.01 a *	0.72 ± 0.10 b	0.49 ± 0.10 a
Clerosterol	0.42 ± 0.15	2.62 ± 0.84 a	1.03 ± 0.56 a	4.33 ± 1.07 b	2.27 ± 0.51 a *
β-Sitosterol	36.60 ± 0.14	54.67 ± 1.10 b	45.05 ± 1.45 a *	51.56 ± 1.02 b	41.42 ± 0.30 a *
Sitostanol	2.22 ± 0.19	2.54 ± 1.58 a	1.93 ± 0.13 a	2.09 ± 0.25 a	2.15 ± 0.17 a
Δ5-Avenasterol	1.54 ± 0.27	3.16 ± 0.68 a	2.41 ± 0.04 a *	2.68 ± 0.03 b	2.04 ± 0.02 a *
Δ5.24-Stigmastadienol	0.49 ± 0.08	1.18 ± 0.39 a	1.54 ± 0.48 a *	nd	0.64 ± 1.11
Δ7-Estigmastenol	5.91 ± 0.03	11.66 ± 1.65 b	8.21 ± 1.26 a *	13.58 ± 0.35 b	9.23 ± 0.09 a *
Δ7-Avenasterol	2.04 ± 0.07	4.31 ± 1.21 a	3.03 ± 0.65 a	4.96 ± 0.19 b	3.16 ± 0.04 a *
Others	0.27 ± 0.04	0.13 ± 0.11 a	0.25 ± 0.03 a	0.15 ± 0.06 a	0.25 ± 0.09 a
Total sterols (mg/kg lipids)	3494 ± 32	2680 ± 66 a	3476 ± 288 b	2333 ± 39 a	2654 ± 50 b *

Abbreviations: HLSO, high-linoleic sunflower oil; HOSO, high-oleic sunflower oil, nd, not detected. Means ± SD (*n* = 3). Different letters in the same row indicate significant differences between the final lipid extract and the final frying oil for a given oil. Asterisks indicate significant differences between the initial and final lipid extracts for a given frying oil (*p* < 0.05).

**Table 9:** Sterols composition and total content of lipids extracted from nuggets and frying oils after the fourth discontinuous frying operation.

Sterols (wt % on oil)	Initial Lipid Extract	HLSO		HOSO	
		Final Oil	Final Lipid Extract	Final Oil	Final Lipid Extract
Cholesterol	61.20 ± 1.40	0.91 ± 0.74 a	52.60 ± 3.21 b *	2.35 ± 2.80 a	55.57 ± 1.10 b *
Campesterol	4,17 ± 0.14	7.78 ± 0.44 b	4.63 ± 0.72 a	8.66 ± 0.32 b	4.57 ± 0.04 a *
Stigmasterol	0.52 ± 0.02	6.63 ± 0.90 b	3.18 ± 0.50 a *	7.48 ± 0.65 b	3.32 ± 0.09 a *
Δ7-Campesterol	2.57 ± 0.09	2.18 ± 0.04 b	1.03 ± 0.16 a *	2.41 ± 0.28 b	1.16 ± 0.07 a *
Δ5.23-Stigmastadienol	0.81 ± 0.03	0.41 ± 0.35 a	0.35 ± 0.16 a *	0.31 ± 0.27 a	0.28 ± 0.07 a *
Clerosterol	0.27 ± 0.02	3.61 ± 1.80 a	1.03 ± 0.88 a *	1.08 ± 1.69 a	0.19 ± 0.12 a *
β-Sitosterol	21.33 ± 0.77	51.18 ± 1.11 b	25.50 ± 3.96 a	53.37 ± 2.31 b	23.89 ± 0.65 a *
Δ5-Avenasterol	1.36 ± 0.03	2.77 ± 1.70 a	1.43 ± 0.31 a	4.43 ± 3.64 a	1.23 ± 0.38 a
Δ5.24-Stigmastadienol	1.17 ± 0.11	3.09 ± 0.63 b	1.62 ± 0.29 a	1.98 ± 1.75 a	1.14 ± 0.04 a
Δ7-Estigmastenol	0.47 ± 0.00	2.99 ± 1.34 b	0.38 ± 0.38 a	1.25 ± 1.42 a	0.68 ± 0.20 a
Δ7-Avenasterol	4.26 ± 0.16	13.25 ± 0.55 b	5.90 ± 0.88 a *	13.15 ± 0.75 b	5.32 ± 0.04 a *
Others	1.68 ± 0.13	4.95 ± 0.07 b	2.29 ± 0.31 a *	3.47 ± 3.01 a	2.15 ± 0.04 a *
Total sterols (mg/kg lipids)	3509 ± 34	3166 ± 98 a	3575 ± 69 b	2854 ± 32 a	3849 ± 80 b *

Abbreviations: HLSO, high-linoleic sunflower oil; HOSO, high-oleic sunflower oil, Means ± SD (*n* = 3). Different letters in the same row indicate significant differences between the final lipid extract and the final frying oil for a given oil. Asterisks indicate significant differences between the initial and final lipid extracts for a given frying oil (*p* < 0.05).

## Data Availability

The original contributions presented in the study are included in the article, further inquiries can be directed to the corresponding author.
